# Successful prediction of a physiological circuit with known connectivity from spiking activity alone

**DOI:** 10.1186/1471-2202-14-S1-P118

**Published:** 2013-07-08

**Authors:** Felipe Gerhard, Tilman Kispersky, Gabrielle J Gutierrez, Eve Marder, Mark Kramer, Uri Eden

**Affiliations:** 1 Brain Mind Institute, Ecole Polytechnique Federale de Lausanne (EPFL), Lausanne, Switzerland; 2 Biology Department and Volen Center, Brandeis University, Waltham, MA, USA; 3 Department of Mathematics and Statistics, Boston University, Boston, MA, USA

## 

Identifying the structure and dynamics of synaptic interactions between neurons is the first step to understanding neural network dynamics. The presence of synaptic connections is traditionally inferred through the use of targeted stimulation and paired recordings or by post-hoc histology. More recently, causal network inference algorithms have been proposed to deduce connectivity directly from electrophysiological signals, such as extracellularly recorded spiking activity. These algorithms have not been validated on a neurophysiological data set for which the actual circuitry is known. Recent work has shown that traditional network inference algorithms based on linear models typically fail to identify the correct coupling of even a basic three-neuron circuit like the crab stomatogastric nervous system.

In this work, we show that point process models of observed spike trains can guide inference of relative connectivity estimates that match the known physiological connectivity of a three-neuron circuit up to a choice of threshold. We elucidate the necessary steps to derive faithful connectivity estimates from a model that incorporates the spike train nature of the data. We then apply the model to measure changes in the effective connectivity pattern in response to two pharmacological interventions, which affect both intrinsic neural dynamics and synaptic transmission.

Our results provide the first successful application of a network inference algorithm to a circuit for which the actual physiological synapses between neurons are known. The point process methodology presented here generalizes well to larger networks and can describe the statistics of neural populations. In general we show that advanced statistical models allow for the characterization of effective network structure, deciphering underlying network dynamics and estimating information-processing capabilities.

